# The IFN-γ/PD-L1 axis between T cells and tumor microenvironment: hints for glioma anti-PD-1/PD-L1 therapy

**DOI:** 10.1186/s12974-018-1330-2

**Published:** 2018-10-17

**Authors:** Jiawen Qian, Chen Wang, Bo Wang, Jiao Yang, Yuedi Wang, Feifei Luo, Junying Xu, Chujun Zhao, Ronghua Liu, Yiwei Chu

**Affiliations:** 1 Department of Immunology, School of Basic Medical Sciences, and Institute of Biomedical Sciences, Fudan University, No. 138, Yi Xue Yuan Rd., Mail Box 226, Shanghai, 200032 People’s Republic of China; 2 Biotherapy Research Center, Fudan University, Shanghai, 200032 China; 3 Jiangsu Key Lab of Medical Optics, Suzhou Institute of Biomedical Engineering and Technology, Chinese Academy of Sciences, Suzhou, 215000 China; 4 Northfield Mount Hermon School, Mount Hermon, MA 01354 USA

**Keywords:** PD-L1, Immune checkpoint, IFN-γ, Glioma, Immune evasion

## Abstract

**Background:**

PD-L1 is an immune inhibitory receptor ligand that leads to T cell dysfunction and apoptosis by binding to its receptor PD-1, which works in braking inflammatory response and conspiring tumor immune evasion. However, in gliomas, the cause of PD-L1 expression in the tumor microenvironment is not yet clear. Besides, auxiliary biomarkers are urgently needed for screening possible responsive glioma patients for anti-PD-1/PD-L1 therapies.

**Methods:**

The distribution of tumor-infiltrating T cells and PD-L1 expression was analyzed via immunofluorescence in orthotopic murine glioma model. The expression of PD-L1 in immune cell populations was detected by flow cytometry. Data excavated from TCGA LGG/GBM datasets and the Ivy Glioblastoma Atlas Project was used for in silico analysis of the correlation among genes and survival.

**Results:**

The distribution of tumor-infiltrating T cells and PD-L1 expression, which parallels in murine orthotopic glioma model and human glioma microdissections, was interrelated. The IFN-γ level was positively correlated with PD-L1 expression in murine glioma. Further, IFN-γ induces PD-L1 expression on primary cultured microglia, bone marrow-derived macrophages, and GL261 glioma cells in vitro. Seven IFN-γ-induced genes, namely *GBP5*, *ICAM1*, *CAMK2D*, *IRF1*, *SOCS3*, *CD44*, and *CCL2*, were selected to calculate as substitute indicator for IFN-γ level. By combining the relative expression of the listed IFN-γ-induced genes, IFN-γ score was positively correlated with PD-L1 expression in different anatomic structures of human glioma and in glioma of different malignancies.

**Conclusion:**

Our study identified the distribution of tumor-infiltrating T cells and PD-L1 expression in murine glioma model and human glioma samples. And we found that IFN-γ is an important cause of PD-L1 expression in the glioma microenvironment. Further, we proposed IFN-γ score aggregated from the expressions of the listed IFN-γ-induced genes as a complementary prognostic indicator for anti-PD-1/PD-L1 therapy.

**Electronic supplementary material:**

The online version of this article (10.1186/s12974-018-1330-2) contains supplementary material, which is available to authorized users.

## Background

Gliomas, characterized by immune evasive hallmarks, are the major primary tumors in the central nervous system (CNS) [1]. The immune microenvironment of glioma is a complex neuroinflammatory network that involves both positive and negative immune regulation [2]. T cells, the main executors in the anti-tumor immune response, are suppressed by various mechanisms at the tumor site [3–6], among which PD-1/PD-L1 axis-mediated functional inhibition plays a key role. PD-L1 is an immune inhibitory receptor ligand expressed on many types of cancer cells, such as melanomas, lymphomas, lung cancers, prostate cancers, and gliomas [7]. By binding to its receptor PD-1 expressed on the surface of activated T cells, PD-L1 leads to T cell dysfunction and apoptosis [8, 9]. This facilitates the immunosuppressive microenvironment and tumor progression. Previously, studies have revealed that PD-L1 upregulation depended on IFN-γ-secreting CD8^+^ lymphocytes [10]. IFN-γ binds with receptor and subsequently activates JAK/STAT signaling pathway, which leads to the downstream expression and activation of IRF-1, further inducing PD-L1 expression on tumor cells [11]. However, the driving factors of PD-L1 expression on various cells in the glioma microenvironment remain to be investigated.

Emerging evidence implies that PD-1/PD-L1 is a promising target to reverse the immune evasion of glioma [12]. Nduom et al. [13] measured PD-L1 expression in 94 patients and found that PD-L1 was a negative prognostic indicator for glioblastoma (GBM). Wang et al. [14] analyzed 976 glioma samples with transcriptome data and concluded that PD-L1 expression was positively correlated with the WHO classification of glioma. While abundant clinical studies on anti-PD-1/PD-L1 antibody specific to gliomas are in progress, the results remain unclear. Based on completed clinical trials of anti-PD-1/PD-L1 therapy targeting other tumors, nevertheless, screening for appropriate patients is crucial for favorable prognosis [15]. Although PD-L1 immunohistochemistry (IHC) has been approved by the FDA as the only predictive companion test for cancer immunotherapy such as pembrolizumab in non-small cell lung cancer patients, supplementary clinical indicators are urgently needed considering the high false negative rate [16]. Until now, biomarkers identifying possible responsive glioma patients have not been defined.

In this study, we investigated the distribution of T cells and PD-L1 expression on murine orthotopic glioma model and validated the results in human glioma samples from databases of the Cancer Genome Atlas (TCGA) and the Ivy Glioblastoma Atlas Project. We found that the distribution of PD-L1 in glioma coincides with morphologically apoptotic T cells and that IFN-γ induced PD-L1 expression on primary cultured microglia, bone marrow-derived macrophages (BMDM), and GL261 tumor cells, suggesting IFN-γ derived from tumor-infiltrating T cells may be the lead to induced PD-L1 expression in the microenvironment. We also found that, apart from the tumor cells previously reported, activated microglia and peripheral-derived macrophages in the microenvironment also present significant upregulation of PD-L1. Considering the importance of IFN-γ in inducing PD-L1 in the glioma microenvironment, it is assumed as a supplementary indicator to predict the expression of PD-L1. However, traditional IHC or RNA seq methods are insufficient to accurately measure the IFN-γ level in tumor samples. Here, we proposed IFN-γ score, aggregated from the expressions of seven IFN-γ-induced genes, as an ancillary marker in screening for appropriate glioma patients.

## Methods

### Mice

C57BL/6 mice (6–8 weeks) were purchased from Shanghai Slac Laboratory Animal Co., Ltd. (Shanghai, China). Mice were maintained under the specific pathogen-free condition and housed in the Animal Facility of Fudan University (Shanghai, China) according to the Guidelines for the Care and Use of Laboratory Animals (No. 55 issued by the Ministry of Health, People’s Republic of China, on January 25, 1998), as administered by the Institutional Animal Care and Use Committee (IACUC) of Fudan University.

### GL261 murine glioma model

GL261 murine glioma cell line was kindly provided by Dr. Liangfu Zhou (Huashan Hospital, Shanghai, China). GL261 was cultured in DMEM/F12 (Thermo Fisher, USA) supplemented with 10% heat-inactivated FBS (Thermo Fisher, USA), 2 mM glutamine (Thermo Fisher, USA), 100 U/ml penicillin (Thermo Fisher, USA), and 100 μg/ml streptomycin (Thermo Fisher, USA). Cells were maintained in the incubator at 37 °C in a humidified 5%CO_2_/95% atmosphere with routine checks for mycoplasma contamination every 3 months. For tumor inoculation, anesthetized mice were immobilized and mounted onto a stereotactic head holder in the flat-skull position. The skin of the skull was dissected in the midline by a scalpel. The skull was carefully drilled with a 20-gauge needle tip (ML + 2.0; RC + 1.0 mm). Then, a microliter Hamilton syringe was inserted to a depth of 3 mm and retracted to a depth of 2.5 mm from the dural surface. Five microliters (2 × 10^4^ cells/μl) of cell suspension or PBS was slowly injected in 2 min. The needle was then slowly taken out from the injection canal, and the skin was sutured. Terminal stage of GL261 murine glioma model was defined by agonal symptoms such as poor grooming, lethargy, weight loss, or seizures.

### Primary adult microglia culture

Microglia were prepared from 6- to 8-week-old mice as described previously [17]. Briefly, the brains were dissected with the cerebella and olfactory bulbs taken off. The tissue was triturated mechanically and washed with PBS by centrifuging for 7 min at 500*g*, 4 °C. The supernatant was discarded, and pellets were re-suspended in 37% Percoll. Percoll gradients (70%/37%/30%/0%) were prepared and centrifuged for 5 min at 500*g*, 18 °C (low acceleration, brake off). Mononuclear cells were collected at 70%/37% Percoll interface. Microglia were enriched by CD11b microbeads (BD Bioscience, USA) according to the manufacturer’s specification and harvested for purity check and further tests. Isolated microglia were plated onto 24-well plates (1 × 10^5^ cells per well) and cultured in basic medium with additional 5 ng/ml recombination TGF-β1 (Miltenyi, Germany) and 10 ng/ml Recombinant Mouse M-CSF Protein (R&D, USA). Half of the medium was changed every 3 days, for a total of 10–14 days.

For T cell co-culture assay, adult microglia were plated onto 96-well plates at a density of 1 × 10^5^ cells per well. Half of the medium was changed every 3 days, for a total of 7 days. On day 8, microglia were treated with or without 20% GCM for 24 h. The CD4^+^ T Cell Isolation Kit (Miltenyi, Germany) was used for purification of CD4^+^ T cells from the spleen of OT II mice. CD4^+^ T cells were stained with CFSE dye (Invitrogen, USA) following the manufacturer’s instructions. The microglia were washed with PBS for three times and then co-cultured with CD4^+^ T cells (4 × 10^5^ cells per well) for 4 days supplied with 0.1 μM OVA323–339 peptides (Sigma-Aldrich, USA). After co-culture, both microglia and T cells were determined by flow cytometric analysis.

### Immunofluorescence

For immunofluorescence, sections were thawed and dried at room temperature and rinsed in PBS. For fixation, cells were washed with PBS and followed by 4% PFA for 5 min. Samples were permeabilized with 0.25% Triton X-100 for 15 min and blocked in blocking buffer containing 10% donkey serum for 2 h at room temperature or overnight at 4 °C. Then, samples were incubated with indicated primary antibodies (Additional file 1: Table S2) overnight at 4 °C. Samples were then washed with PBS and incubated with the appropriate fluorophore-conjugated secondary antibodies, namely Alexafloure-488, 594 (Thermo Fisher, USA) and Cy3 (JacksonImmunoResearch Laboratory, USA), at a dilution of 1:500 in 1% BSA for 1 h at room temperature. 4′, 6-Diamidino-2-phenylindole (DAPI) was used as a counterstain. Images were acquired by a fluorescence microscope Olympus IX73 (Olympus, Japan). Appropriate gain and black level settings were determined by control tissues stained with secondary antibodies. Analyses of images were performed using ImageJ software (NIH, USA). Quantitative analysis was performed with ImageJ to determine the T cell counts and mean intensity of PD-L1. For CD4^+^ cell or CD8^+^ cell counts, data were collected from five random fields for each region per mouse, *n* = 4. For mean intensity of PD-L1, data were collected from at least three and up to seven random fields for each region per mouse, *n* = 5.

### FACS analyses

For fluorescence-activated cell sorting (FACS) analysis of brain tumor-infiltrating immune cells, mice were euthanized at the defined endpoint. Mononuclear cells in the brains were isolated as previously described and stained afterward with the respective antibodies for FACS analysis. For flow cytometry, cells were counted and incubated with Fc blocker (eBiosciences, USA) for 30 min, followed by another 30-min incubation with conjugated antibodies for extracellular markers. For intracellular cytokine detection, cells were stimulated in vitro with Cell Stimulation Cocktail (eBiosciences, USA) for 5 h at 37 °C before FACS analysis. After stimulation, cells were stained for surface markers and cytokines with Intracellular Fixation and Permeabilization Buffer Set (eBiosciences, USA). All antibodies used for these experiments were listed in Additional file 1: Table S2. Proper isotype controls and compensation controls were performed in parallel. BD Biosciences Canto II (BD Biosciences, USA) was used for flow cytometry. FlowJo software (Tree Star, USA) was used for FACS data analysis.

### Quantitative real-time PCR

Total RNA was isolated with RNAiso (Takara, Japan) following the manufacturer’s protocol and reversely transcribed using PrimeScript™ RT reagent Kit with gDNA Eraser (Perfect Real Time) (Takara, Japan). Gene expression was detected using SYBR® Premix Ex TaqTM II (Tli RNaseH Plus) Kit (Takara, Japan). All RT-PCR amplifications were performed in triplicates in a 20-μl reaction volume with the indicated primer pairs. Primer sequences were listed in Additional file 2: Table S1. RT-PCRs were performed using 7500 Fast Real-Time PCR System (Applied Biosystems, USA). The amount of target mRNA was normalized to the expression level of β-actin generated from the same sample and subsequently to controls. Relative expression was calculated as 2^−ΔCt^.

### IFN-γ score calculation and clinical data analysis

Firstly, 34 genes were sorted out by filtering genes from GO term: response to interferon-gamma (accession GO: 0034341, organism: *Homo sapiens*) with genes that were positively correlated with PD-L1 expression (*p* < 0.05; *r* > 0.5) from the TCGA lower grade glioma (LGG)/GBM datasets. Then, further crossing 34 genes with 840 genes that were positively correlated with PD-L1 expression (*p* < 0.05; *r* > 0.3) from the Ivy Glioblastoma Atlas Project, 7 genes were eventually sorted out, namely *GBP5*, *ICAM1*, *CAMK2D*, *IRF1*, *SOCS3*, *CD44*, and *CCL2.* Combining the relative expression levels of the sorted seven genes, IFN-γ score was calculated as a substitute indicator for IFN-γ level. Myeloid cell-related genes (*CD14*, *CD33*, *CD36*, *CD68*, *CX3CR1*, *ENG*, *ITGAL*, and *ITGAM*) were used to calculate myeloid cell score. T cell-related genes (*CD2*, *CD3D*, *CD3E*, *CD3G*, *CD4*, *CD8A*, *CD8B*, *CD28*, *CCR7*, and *IL2RA*) were used to calculate T cell score. The median value of IFN-γ score was used as the cutoff to divide patients with high IFN-γ score and patients with low IFN-γ score.

### Data presentation and statistical analysis

GraphPad Prism 6.0 (GraphPad Software Inc., USA) was used for all data analysis. Parametric data were presented as mean ± standard error of the mean (SEM). Differences between two groups were analyzed using Student’s unpaired *t* test. Analysis of variance (ANOVA) was used to compare multiple groups, and Pearson’s correlation coefficient was used to analyze the correlation of the expression levels of genes. Statistical significance was determined at *p* < 0.05 in all cases.

## Results

### PD-1 expression on T cells was upregulated during glioma progression

**Fig. 1:**
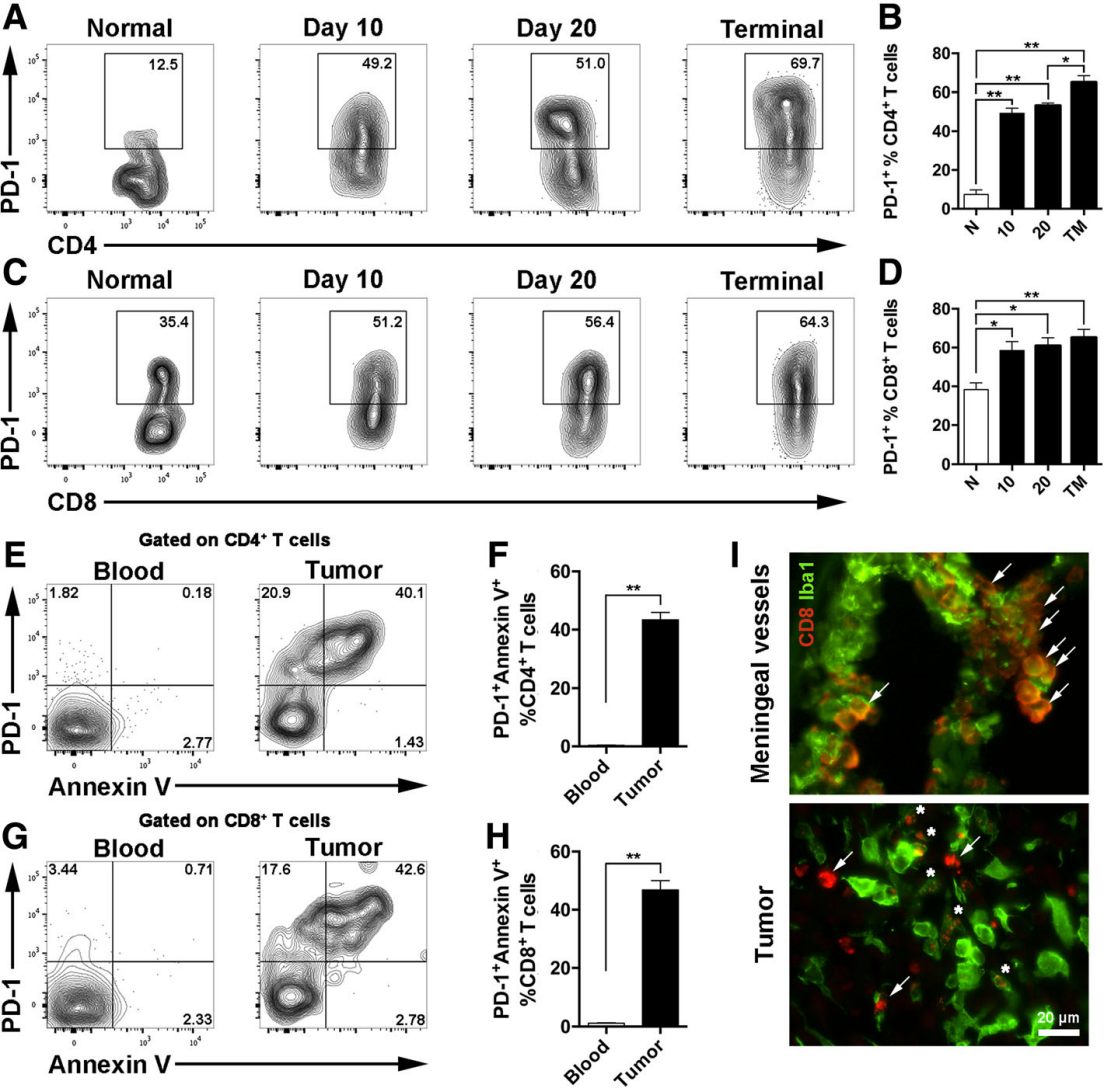
PD-1 expression on T cells was upregulated during glioma progression that correlate with apoptosis. **a**–**d** Flow cytometry analysis of PD-1 expression on glioma-infiltrating T cells at indicated time points. Representative data of the PD-1 expression of CD4^+^ T cells (**a**) and CD8^+^ T cells (**c**) at the indicated time point and the statistical summary for CD4^+^ T cells (**b**) and CD8^+^ T cells (**d**), *n* = 3–4. N, normal brain; 10, 10 days after tumor inoculation; 20, 20 days after tumor inoculation; TM, terminal stage. **e**–**h** Flow cytometry analysis of T cell apoptosis by annexin V. Representative data of the apoptosis level of CD4^+^ T cells (**e**) and CD8^+^ T cells (**g**) from the peripheral blood and glioma tissue. The statistical summary for CD4^+^ T cells (**f**) and CD8^+^ T cells (**h**), *n* = 12. **i** Representative staining for Iba1 (green) and CD8 (red) in tumor-bearing brain (day 20). Arrow, morphologically intact T cells; asterisk, disintegrated T cells. Scale bar, 20 μm. One-way ANOVA was performed in **b** and **d**. Unpaired Student’s *t* test was performed in **f** and **h**. *p < 0.05; **p < 0.01. All values are shown as mean ± SEM

We investigated the expression of PD-1 in tumor-infiltrating T cells during tumor progression in GL261 glioma orthotopic murine glioma model, the survival time of which is around 30 days [18]. Flow cytometry analysis of glioma-infiltrating immune cells showed that PD-1 expression on both CD4^+^ and CD8^+^ T cells gradually increased as tumor progressed (Fig. 1a–d), and non-inoculated brain was used as normal tissue. PD-1 was highly expressed on tumor-infiltrating T cells, and annexin V labeling revealed PD-1 expression was positively correlated with apoptosis of T cells, while the corresponding peripheral blood-derived T cells presented low PD-1 expression and no tendency to apoptosis (Fig. 1e–h). Besides, according to immunofluorescence, tumor-infiltrating T cells presented typical apoptotic morphology, such as reduced size compared with non-apoptotic CD8^+^ T cells in meningeal vessels. Moreover, there were apoptotic bodies bounded by CD8^+^ membrane and phagocytized by neighboring Iba1^+^ microglia/macrophages. T cells accumulated at the meninges were in typical T cell shape and morphologically normal (Fig. 1i). The above suggests that upregulated PD-1 expression on T cells is related to its apoptosis at the glioma tumor site and the tumor microenvironment causes PD-1 induction in the T cells.

### The distributions of tumor-infiltrating T cells and PD-L1 in the glioma microenvironment were interrelated

**Fig. 2:**
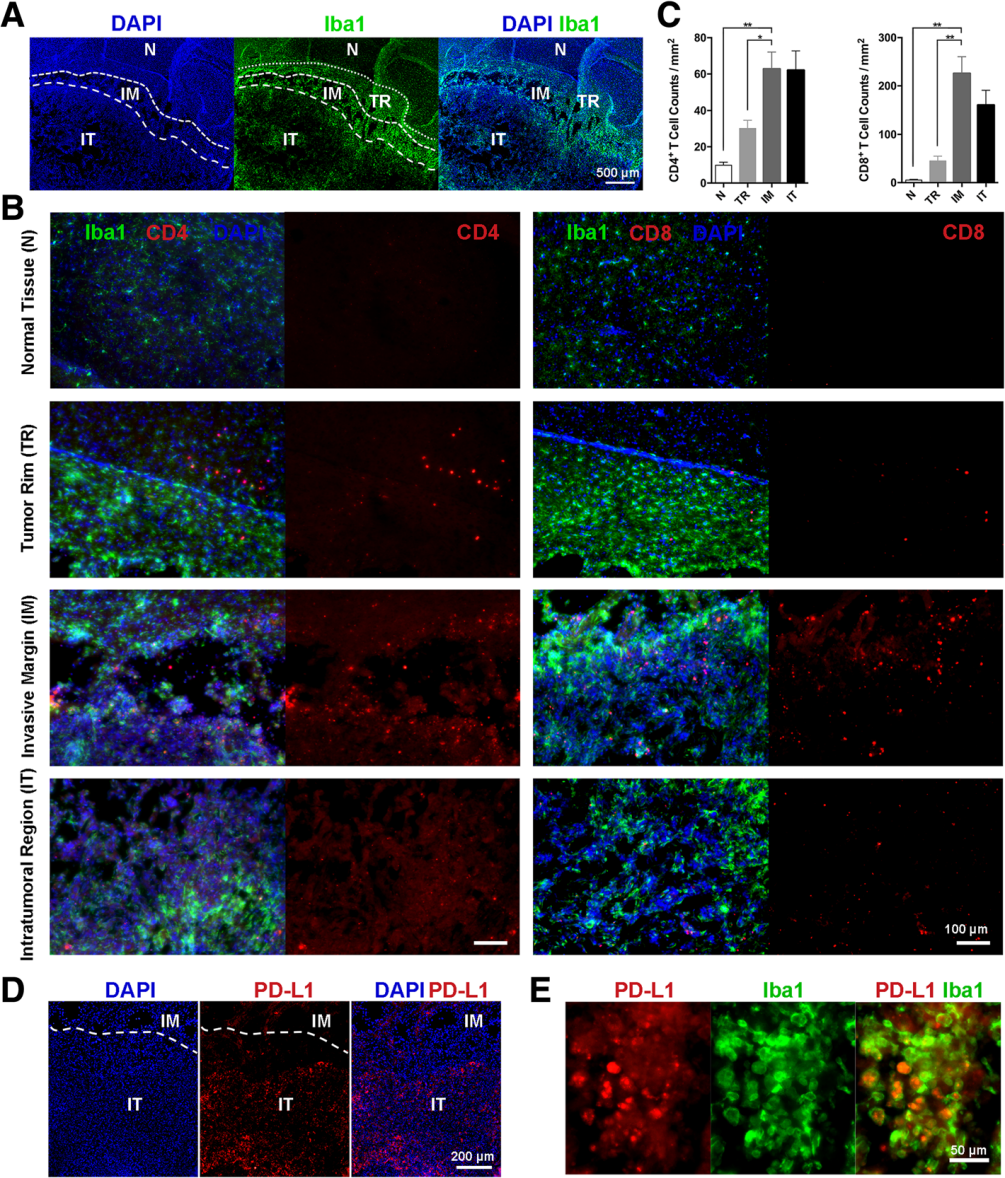
The distribution of tumor-infiltrating T cells and PD-L1 in murine glioma model. **a** Murine glioma is divided into four parts according to the density and morphological characteristics of Iba1+ myeloid cells. Representative staining for Iba1 (green) and DAPI (blue) in the tumor-bearing brain (day 20). N, normal tissue; TR, tumor rim; IM, invasive margin; IT, intratumoral region. Scale bar, 500 μm. **b** Representative staining for Iba1 (green), CD4 (red) or CD8 (red), and DAPI (blue) in the tumor-bearing brain (day 20); different regions of the tumor-bearing brain were shown. Scale bar, 100 μm. **c** CD4^+^ cell or CD8^+^ cell counts per field were calculated. Data were collected from five random fields for each region per mouse, *n* = 4. One-way ANOVA was performed. *p < 0.05; **p < 0.01. All values are shown as mean ± SEM. **d** Representative staining for PD-L1 (red) and DAPI (blue) in the tumor-bearing brain (day 20). Scale bar, 200 μm. **e** Representative staining for Iba1 (green) and PD-L1 (red) in the IT area of tumor-bearing brain (day 20). Scale bar, 50 μm

We then analyzed the distribution of tumor-infiltrating T cells. According to the density and morphological characteristics of Iba1^+^ myeloid cells, the tumor area is divided into four parts. They are normal tissue (N), where microglia were of low density and in a typical ramified shape with small soma; tumor rim (TR), where microglia were of increased density with thicker branches and enlarged soma; invasive margin (IM), where a large number of microglia gathered at the leading edge of tumor invasion; and intratumoral region (IT), where Iba1^+^ myeloid cells were typically amebiform (Fig. 2a). Immunofluorescence analysis of T cell density in each tumor area indicated that IM and IT were the major infiltrating areas of T cells (Fig. 2b, c). While T cells of high density in the IM area were morphologically activated, those in the IT area were morphologically apoptotic. Besides, many CD4^+^ or CD8^+^ apoptotic bodies can be recognized in the IT area (Figs. 1i and 2b). In accordance with the apoptotic status of T cells in the IT area, PD-L1 expression was mainly found in the same IT area, especially around the necrotic tissue (Fig. 2d). Immunofluorescence analysis of PD-L1 intensity in each tumor area also indicated that IM and IT showed comparatively high PD-L1 expression, while N and TR had almost no expression of PD-L1 (Additional file 3: Figure S1A, B). Together, tumor-infiltrating T cells and PD-L1 presented unique distribution patterns in the tumor microenvironment. Different status of T cells can be found in tumor areas with high T cell density, which agrees with the expression of PD-L1 in the corresponding area. We speculate that, although successfully infiltrated in the tumor microenvironment, T cells are soon rendered inactive and even apoptotic, because of the upregulated PD-1 and its binding with high expression of PD-L1 in the certain tumor area.

Previous studies mainly focused on the expression of PD-L1 on tumor cells and its role in CTL inhibition, and PD-L1 expression on APCs and its potential role in the dysfunction of CD4^+^ T cells remain neglected. Besides, our previous work demonstrated that most of the brain resident APCs, Iba1^+^ microglia, were mainly presented in the IM and IT regions [17]. Immunofluorescence assay manifested likewise that Iba1^+^ myeloid cells highly expressed PD-L1 in the IT area (Fig. 2e).

### PD-L1 expression was found on glioma-infiltrating macrophages and microglia

**Fig. 3:**
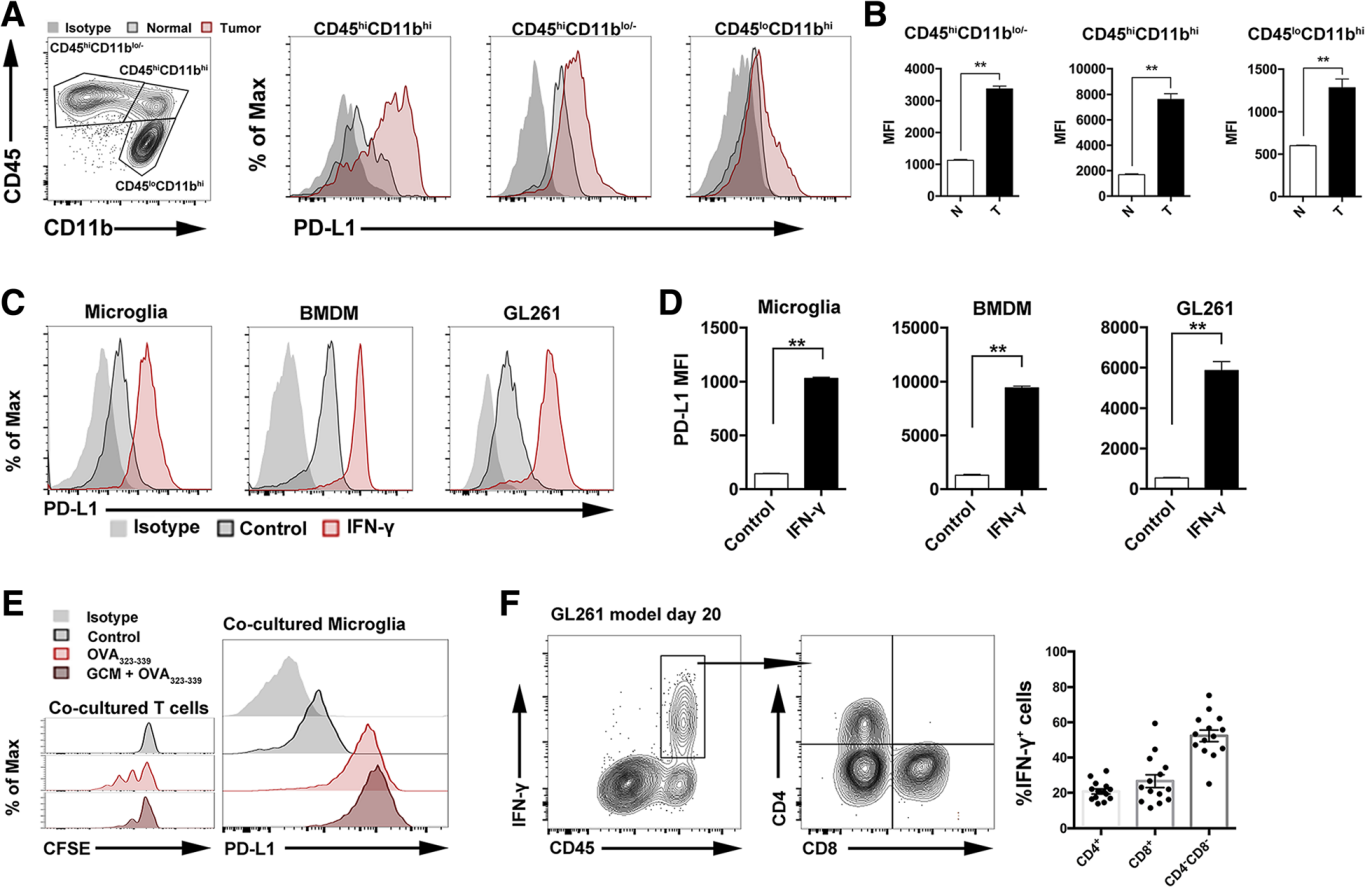
IFN-γ correlated with the upregulation of PD-L1 in glioma microenvironment. **a** The gating strategies of flow cytometric analysis and the analysis of PD-L1 expression on the glioma-infiltrated immune cells. Tumor-infiltrated cells were isolated from GL261 model (day 20). **b** The statistical summary for PD-L1 expression on the major glioma-infiltrated immune cells, *n* = 6. **c** Primary microglia, BMDM, or GL261 cell line treated with IFN-γ (20 ng/ml) for 24 h, and the PD-L1 expression was analyzed by flow cytometry. **d** The statistical summary for PD-L1 expression of the cells, *n* = 3. **e** Microglia were treated with or without GCM (20%, vol/vol) for 24 h. Then, microglia were washed with PBS after stimulation. OT II mice-derived CD4^+^ T cells were isolated and stained with CFSE dye. Microglia and CD4^+^ T cells were co-cultured for 4 days supplied with or without OVA_323–339_ peptides (0.1 μM). Flow cytometry analysis of the co-cultured microglia for PD-L1 level. **f** Flow cytometric analysis of tumor-infiltrating IFN-γ^+^ cells. Tumor-infiltrated cells were isolated from the GL261 model (day 20). Right panel, the summary of IFN-γ^+^ cell population. Unpaired Student’s *t* test was performed in **b** and **d**. **p < 0.01. All values are shown as mean ± SEM

We next analyzed the expression of PD-L1 in the major immune cell populations in the glioma microenvironment. We found that, compared with the CD45^hi^CD11b^lo/−^ subsets and CD45^lo^CD11b^hi^ subsets, PD-L1 was highly expressed in a population of CD45^hi^CD11b^hi^ myeloid cells, the majority of which were activated microglia and peripheral-derived monocytes/macrophages (Fig. 3a, b). Together, PD-L1 is highly expressed in activated microglia and infiltrating myeloid cells, which might also account for T cell dysfunction and apoptosis.

### IFN-γ correlated with upregulation of PD-L1 in the glioma microenvironment

The areas with high PD-L1 expression had simultaneously a large number of infiltrating T cells. It is known that IFN-γ induces PD-L1 expression on many cell types including glioma cell lines in vitro [19, 20]. We stimulated primary cultured microglia, BMDM, and GL261 tumor cells with IFN-γ in vitro, and flow cytometry analysis showed dramatic upregulation of PD-L1 on all three types of cells (Fig. 3c, d). Through microglia-mediated T cell proliferation activation experiments, we found that the PD-L1 expression of microglia significantly upregulated in the OVA_323–339_- and GCM/OVA_323–339_-treated groups (Fig. 3e). Notably, regardless of the level of T cell proliferation, as long as T cells were activated, they could significantly upregulate PD-L1 expression in antigen-presenting cells. The above results suggest that IFN-γ plays a major role in PD-L1 expression in the glioma microenvironment. Based on the flow cytometry analysis that the IFN-γ-secreting cells in GL261 tumors were T cells and CD45^hi^ CD4^−^ CD8^−^ NK subsets (Fig. 3f), we speculate that T cells and NK cells are the major source of IFN-γ in the tumor microenvironment.

### Distribution pattern of tumor-infiltrating T cells and PD-L1 in human glioma samples

**Fig. 4:**
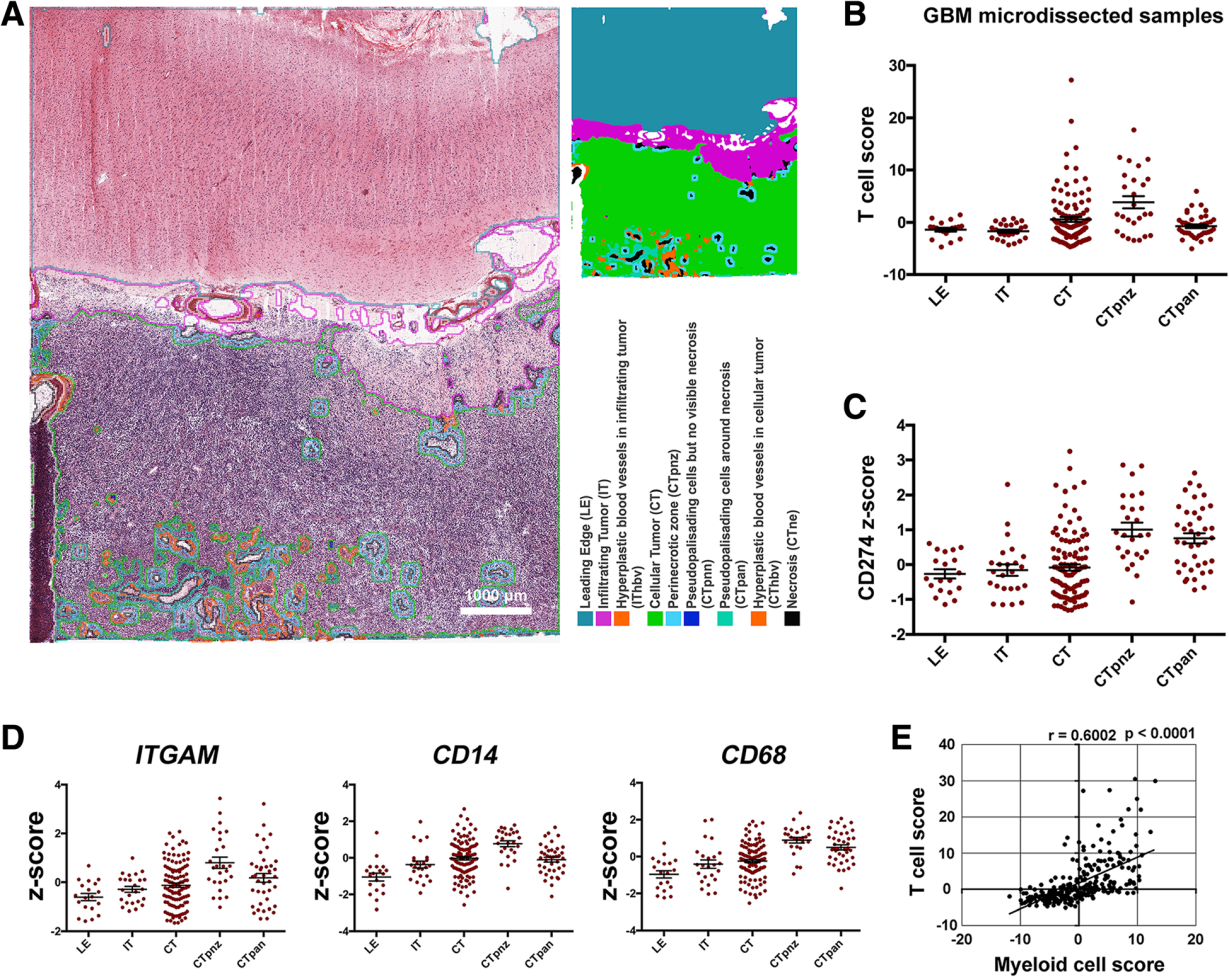
Distribution pattern of tumor-infiltrating T cells and PD-L1 in human glioma samples. **a** Tumor feature annotation of human glioma sample in the Ivy Glioblastoma Atlas Project. Scale bar, 1000 μm. Image credit: Allen Institute. T cell score (**b**) and PD-L1 expression (**c**) in different parts of human glioma samples, *n* = 19–111. LE, leading edge; IT, infiltrating tumor; CT, cellular tumor; CTpnz, perinecrotic zone; CTpan, pseudopalisading cells around necrosis. **d** The expression of myeloid cell signature genes in different parts of human glioma samples according to the Ivy Glioblastoma Atlas Project, *n* = 19–111. **e** Correlation analysis between T cell score and myeloid cell score in human glioma samples. The data were derived from the Ivy Glioblastoma Atlas Project. Pearson’s correlation coefficient was performed

Similar distribution patterns of T cells and PD-L1 expression were found in human glioma samples as well, based on the data from the Ivy Glioblastoma Atlas Project (Fig. 4a). According to the expression level of T cell-related genes, T cells are mainly located in the cellular tumor (CT) area and perinecrotic zone (CTpnz) in human glioma samples (Fig. 4b), which were parallel to the IM and IT area in the murine model. PD-L1 expression in human glioma samples, on the other hand, was found mainly at the region of CTpnz and pseudo palisading cells around necrosis (CTpan) (Fig. 4c), which were equivalent to the area around the necrotic tissue. These data confirm that the expression pattern of PD-L1 and T cells in human samples are likewise consistent with those in the murine glioma model, supporting the correlation between PD-1/PD-L1 axis and glioma-infiltrating T cell apoptosis. In addition, according to the database of the Ivy Glioblastoma Atlas Project, the distribution of PD-L1 was consistent with the distribution of the myeloid gene markers such as *ITGAM*, *CD14*, and *CD68* (Fig. 4c, d). Moreover, T cell score and myeloid cell score were positively correlated in human glioma samples (Fig. 4e).

### IFN-γ-induced genes were positively correlated with progression of glioma and PD-L1 expression

While anti-PD-1/PD-L1 immune checkpoint blockade therapy serves as a promising glioma treatment with good prospect, it is still controversial to use the expression level of PD-L1 as a prognostic indicator for GBM. Certain limitations exist if PD-L1 is used alone as the indicator to screen patients suitable for PD-1/PD-L1 antibody treatment. Considering the apparent heterogeneity of PD-L1 distribution, tumor deemed PD-L1 negative might actually be PD-L1 positive at another biopsy site. A single slide from a single biopsy site is obviously a lack of representativeness. IFN-γ, correlated with upregulation of PD-L1, acts as an efficient cytokine and is expressed by only a few activated lymphocytes in the tumor, making it suitable for prognostic indicator. However, traditional IHC or RNA seq methods are insufficient to accurately measure the IFN-γ level in tumor samples. Therefore, we proposed IFN-γ score as a synergistic marker that could be used to predict PD-L1 expression in glioma samples.

**Fig. 5:**
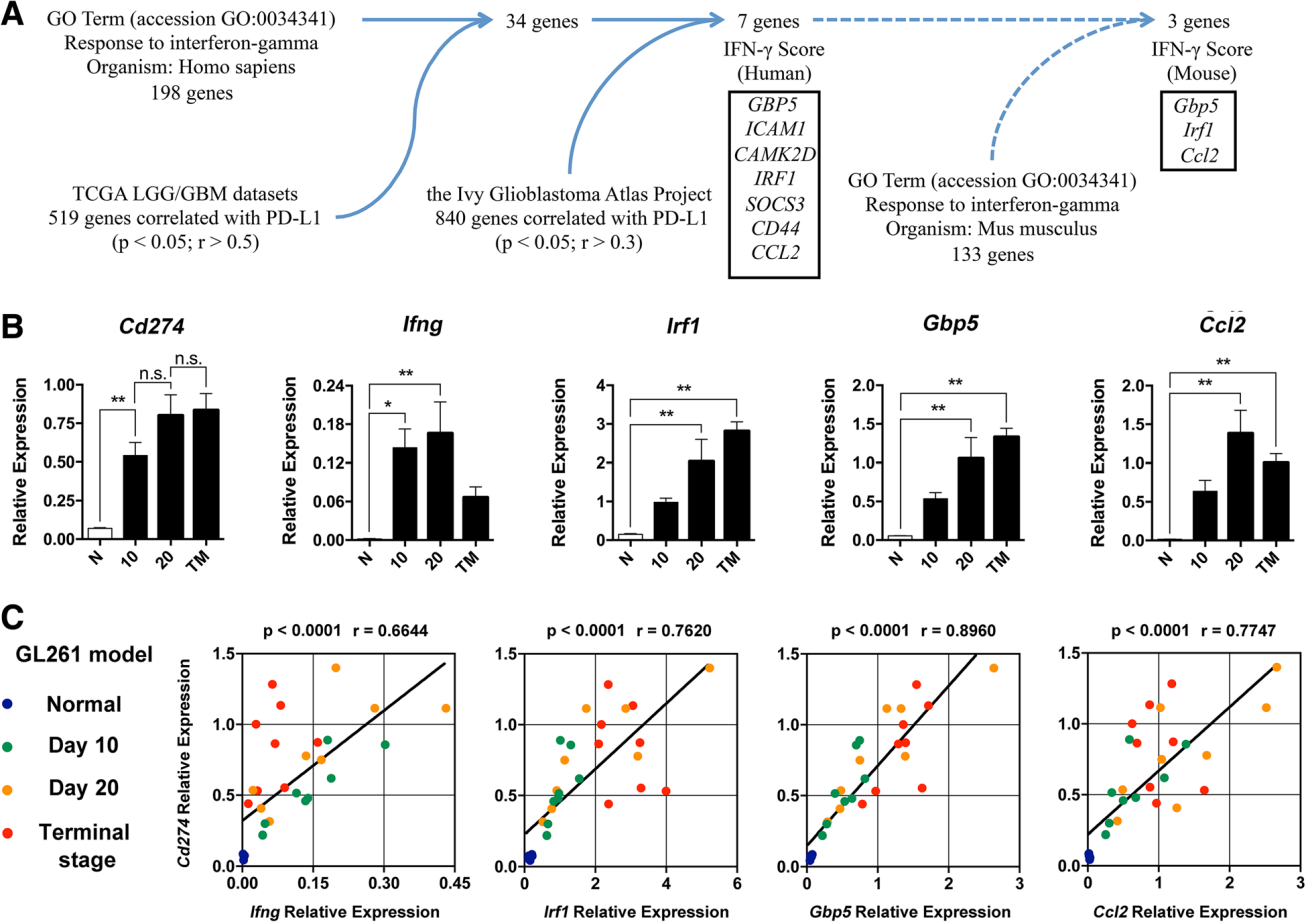
IFN-γ-induced genes are positively correlated with progression of glioma and PD-L1 expression. **a** The schematic figure of selection strategy for genes to calculate IFN-γ score in mouse. **b** The statistical summary for the expression of *Cd274*, *Ifng*, *Irf1*, *Gbp5*, and *Ccl2* in different progression stages of murine GL261 glioma, *n* = 8. **c** Correlation analysis of the expression of *Cd274* with *Ifng*, *Irf1*, *Gbp5*, and *Ccl2* in different progression stages of murine GL261 glioma. One-way ANOVA was performed in **b**. Pearson’s correlation coefficient was performed in **c**. *p < 0.05; **p < 0.01. All values are shown as mean ± SEM

To find the substitute indicator for IFN-γ level, we looked through the IFN-γ-induced genes. A total of 34 genes were sorted out by filtering 198 genes from GO term: response to interferon-gamma (accession GO: 0034341, organism: *Homo sapiens*) with 356 genes that were positively correlated with PD-L1 expression (*p* < 0.05; *r* > 0.5) from the TCGA lower grade glioma (LGG)/GBM datasets. Further crossing these 34 genes with 840 genes that were positively correlated with PD-L1 expression (*p* < 0.05; *r* > 0.3) from the Ivy Glioblastoma Atlas Project, 7 genes were eventually sorted out, namely *GBP5*, *ICAM1*, *CAMK2D*, *IRF1*, *SOCS3*, *CD44*, and *CCL2* (Fig. 5a). Based on the TCGA LGG/GBM datasets, the expression of each listed gene is positively correlated with the malignancy degree of glioma (Additional file 4: Figure S2A) and negatively with the survival of patients (Additional file 4: Figure S2B). By crossing these 7 genes with 133 genes from GO term: response to interferon-gamma (accession GO: 0034341, organism: *Mus musculus*), *Gbp5*, *Irf1*, and *Ccl2* were selected for further verification in the murine glioma model (Fig. 5a). According to qPCR, the relative expression of these three genes were low in the normal mice and increased as glioma progressed, which agreed with the relative expression of *Cd274* (PD-L1) and *Ifng* (Fig. 5b). Moreover, the expression of *Cd274* was well correlated with the respective expression of *Ifng*, *Irf1*, *Gbp5*, and *Ccl2* (Fig. 5c), demonstrating that selected IFN-γ-induced genes serve as feasible substitute indicators for IFN-γ level and thus might synergistically indicate the prognosis of glioma.

### IFN-γ score: a candidate for prognostic indicator of glioma

**Fig. 6:**
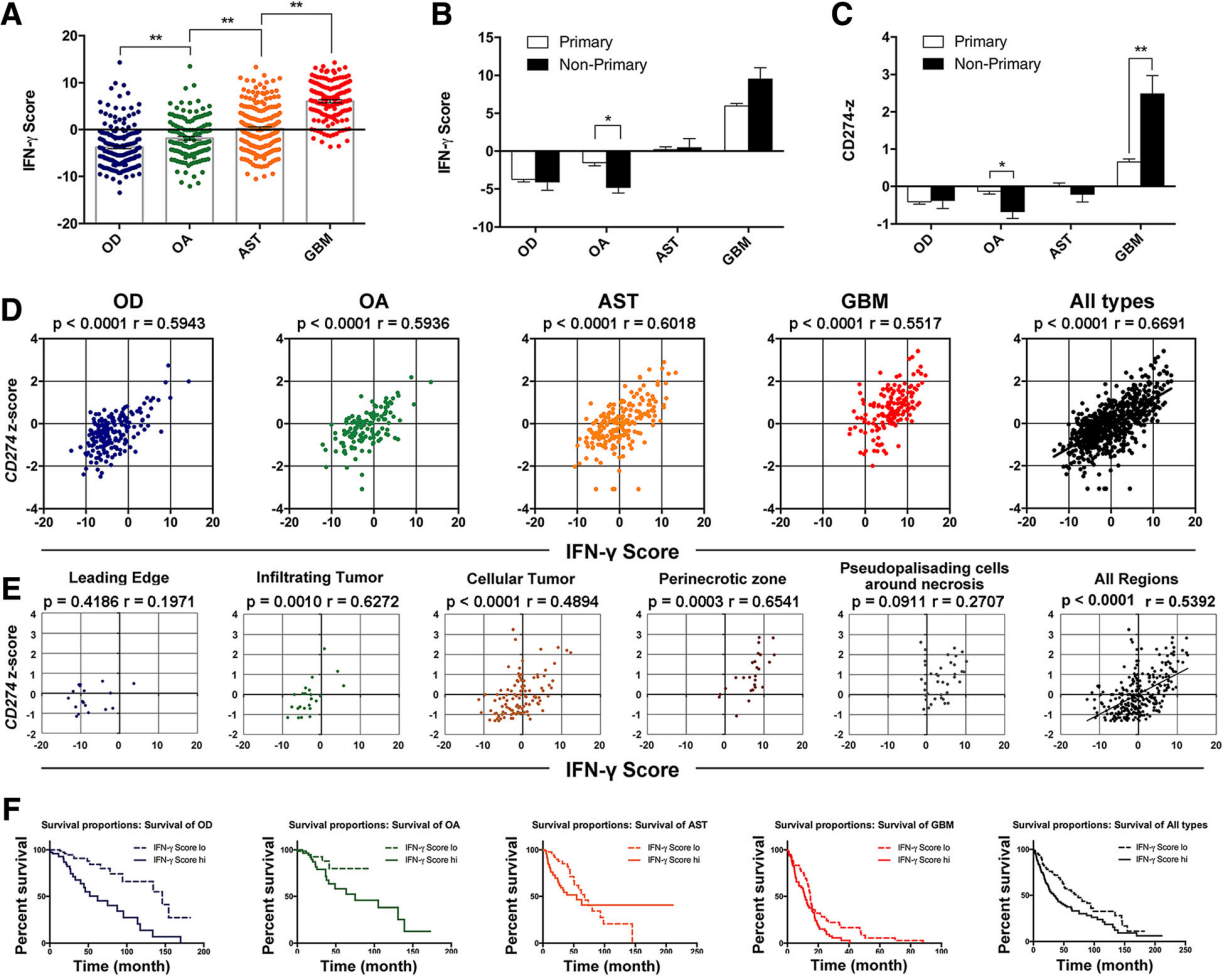
IFN-γ score is an efficient candidate for prognostic indicator of glioma (**a**) IFN-γ score increases along with the malignancy degree of glioma based on the LGG/GBM TCGA datasets. OD, oligodendroglioma; OA, oligoastrocytoma; AST, astrocytoma; GBM, glioblastoma multiforme. The IFN-γ score (**b**) and *CD274* (**c**) presented a similar pattern in both primary and non-primary glioma of various malignancies. **d** The IFN-γ score was correlated with the expression of PD-L1 (*CD274*) based on the LGG/GBM TCGA datasets. **e** Correlation analysis between IFN-γ score and PD-L1 expression in different parts of human glioma samples. The data were derived from the Ivy Glioblastoma Atlas Project. **f** The IFN-γ score was negatively correlated with the survival of glioma patients based on the LGG/GBM TCGA datasets. Unpaired Student’s *t* test was performed in **b** and **c**. Pearson’s correlation coefficient was performed in **d** and **e**. *p < 0.05; **p < 0.01. All values are shown as mean ± SEM

Combining the relative expression levels of the seven sorted genes, IFN-γ score was calculated as substitute indicator for IFN-γ level. Based on TCGA LGG/GBM datasets, the IFN-γ score increased along with the malignancy degree of glioma, reaching an extremely high value in GBM (Fig. 6a; Additional file 4: Figure S2A). The IFN-γ score presented a similar pattern as the expression levels of PD-L1 in both primary and non-primary glioma of various malignancies (Fig. 6b, c). IFN-γ score was positively correlated with the expression of PD-L1 in different types of glioma samples (Fig. 6d). Positive correlation of IFN-γ score and the expression of PD-L1 was also found in different anatomic structures of glioma, based on the Ivy Glioblastoma Atlas Project database (Fig. 6e). In addition, IFN-γ score was negatively correlated with the survival of glioma patients (Fig. 6f; Additional file 4: Figure S2B). All of the above confirmed the feasibility of IFN-γ score as complementary indicator for prognosis of glioma patients.

**Fig. 7:**
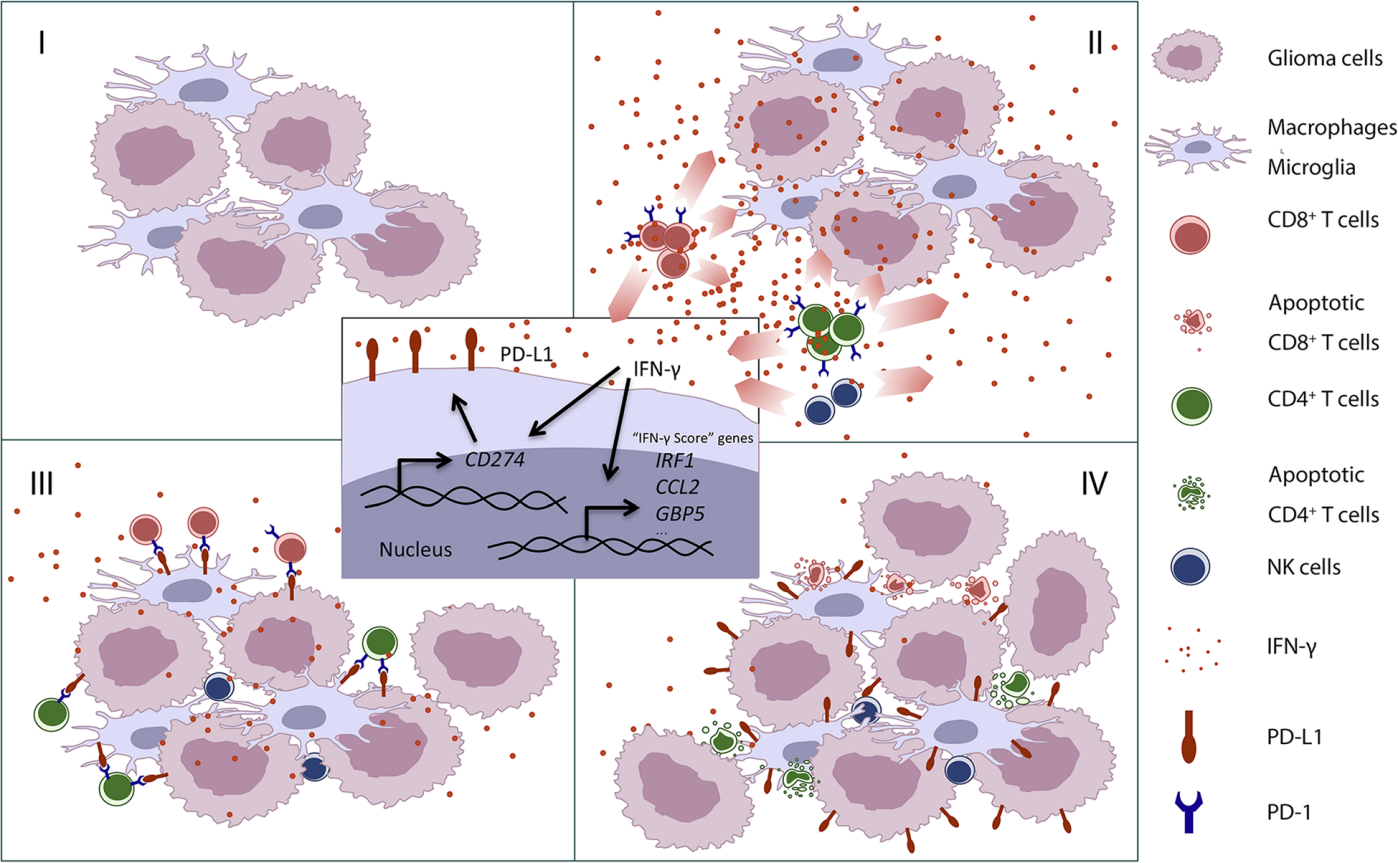
Working model for the mechanism of IFN-γ-induced upregulation of PD-L1 in the glioma microenvironment. Tumor-infiltrating T cells are initially activated and upregulate the expression of PD-1. IFN-γ, secreted by activated T cells and possibly NK cells, induces the expression of PD-L1 not only on tumor cells, but also on microglia and peripheral infiltrating immune cells. Through PD-L1/PD-1 axis, tumor-infiltrating T cells are rendered dysfunctional and apoptotic. Here, we propose IFN-γ score aggregated from seven IFN-γ-induced genes, namely *GBP5*, *ICAM1*, *CAMK2D*, *IRF1*, *SOCS3*, *CD44*, and *CCL2*, as auxiliary prognostic indicator for screening suitable patient for anti-PD-1/PD-L1 therapy

In conclusion, tumor-infiltrating T cells are initially activated and upregulate the expression of PD-1. IFN-γ, secreted by activated T cells and possibly NK cells, induces the expression of PD-L1 not only on tumor cells but also on microglia and peripheral infiltrating immune cells. Through PD-L1/PD-1 axis, tumor-infiltrating T cells are rendered dysfunctional and apoptotic. Here, we propose IFN-γ score aggregated from seven IFN-γ-induced genes, namely *GBP5*, *ICAM1*, *CAMK2D*, *IRF1*, *SOCS3*, *CD44*, and *CCL2*, as auxiliary prognostic indicator for screening suitable patients for anti-PD-1/PD-L1 therapy (Fig. 7).

## Discussion

Our study identified the distribution of PD-L1 in gliomas and that, apart from tumor cells in the tumor microenvironment, significantly increased PD-L1 expression was also spotted on activated microglia and peripheral-derived myeloid cells. Besides, we provided some evidence that IFN-γ played an important role in inducing the expression of PD-L1 in gliomas. IFN-γ score, aggregated from expression of IFN-γ downstream genes as a substitute for the abundance of IFN-γ, is expected to serve as an auxiliary prognostic indicator for screening potential PD-1/PD-L1 antibody drug-applicable glioma patients.

Previous studies have focused on the mechanisms of PD-L1 expression in tumor cells, which include tumor endogenous proto-oncogenic signal, such as abnormal PI3K/Akt signaling pathway [21], and adaptive immune resistance, specifically the magnified negative feedback of the immune system that originally prevents over-activated immune cells from damaging the tissue [22, 23]. In gliomas, the latter mechanism may play a greater role in the expression of PD-L1 in the microenvironment. T cells are activated in the local region of tumor and thus secrete IFN-γ [24–26], which can subsequently induce upregulation of PD-L1 in tumor cells and immune cells in the microenvironment [11, 27], thereby inhibiting tumor eradication led by T cells. Notably, IFN-γ in the tumor microenvironment comes not only from T cells but also from NK cells. The vicious effect of this negative feedback may be more pronounced in the CNS. The microglia, astrocytes, neurons, and epithelial cells in the CNS can be induced by IFN-γ and upregulate PD-L1 expression [28–30], which may exacerbate T cell dysfunction and apoptosis in gliomas. Such IFN-γ/PD-L1 axis-mediated immune suppression that also exists in the normal tissue inadvertently promotes glioma immune escape.

In the GL261 glioma model, we found that the expression of PD-1 was elevated in both CD4^+^ and CD8^+^ T cells. Previous studies have revealed that PD-L1 expressed by tumor cells suppresses cytotoxic activity of tumor-infiltrating CTLs [31–33]. In addition, we found that glioma-infiltrated antigen-presenting cells (microglia and peripheral-derived macrophages) overexpressed PD-L1. The abovementioned suggests the importance of PD-1/PD-L1 axis on the functional inhibition of CD4^+^ cells in glioma. It is indicated that the adaptive immune resistance not only occurs as the inhibition of CTL-mediated tumoricidal activity, but also as the activation of CD4^+^ helper T cells in tumors, thus fundamentally affecting the entire tumor-immune microenvironment networks and disrupting the formation of anti-tumor immune microenvironments.

In the study, we proposed IFN-γ score as a complementary predictive biomarker, which is aggregated from the expression of seven selected genes. IRF1, directly participating in the regulation of PD-L1 expression, is essential in the constitutive and IFN-γ-induced expression of PD-L1 in various cancer cells [34–36]. A recent study confirmed that PD-L1 expression in melanoma cells is mainly regulated by the IFN-γ receptor signaling pathway which subsequently converged to the binding of IRF1 with the PD-L1 promoter [11]. Upregulation of GBP5 has been recognized in colon cancer [37]. In gastric cancer, positive correlation between immune cell infiltration and stromal and epithelial GBP5 expression has been reported [38]. A previous study demonstrated the role of GBP protein in the formation of inflammasome complex as well as its anti-inflammatory and autoimmunity-controlling effect [39], yet the specific function of the GBP protein family remains to be discovered. SOCS3, promoted by IFN-γ downstream genes STAT1 or STAT3 [40, 41], is known as a negative regulator of cytokine signaling and participant in control of CNS immunity [42, 43]. CCL2 is the key chemokine that recruits myeloid-derived cells to the tumor microenvironment in glioma [44]. According to previous studies, peripherally derived myeloid cells usually perform immunosuppressive functions in gliomas [45–47]. Besides, the expression levels of all seven genes were negatively correlated with the survival of glioma patients. All the evidence indicated that the expression of PD-L1 and other immune inhibitory mechanisms in the glioma microenvironment might serve as negative feedback mechanisms that followed, rather than preceded, T cell activation and IFN-γ secretion.

## Conclusions

Our study proposed a possibility that the expression of PD-L1 in the glioma microenvironment is intrinsically driven by the immune system and implies that anti-PD-1/PD-L1 therapy might be preferentially beneficial for patients with high IFN-γ score. More importance should be attached to the screening of potentially responsive patients. Further investigation is required to examine the IFN-γ score and the response to anti-PD-1/PD-L1 therapy in clinical trials.

## Additional files

Additional file 1: **Table S2.** Technical specifications of antibodies used in our study. (DOC 46 kb)

Additional file 2: **Table S1.** Primer sequences for qPCR used in the study. (DOC 31 kb)

Additional file 3: **Figure S1.** Distribution of PD-L1 in the murine glioma model. (A) Representative staining for PD-L1 (red) and DAPI (blue) in the tumor-bearing brain (day 20); different regions of the tumor-bearing brain were shown. Scale bar, 100 μm. (B) PD-L1 intensity of different regions was calculated. Data were collected from four to seven randomly selected areas for each corresponding region per mouse, *n* = 3. One-way ANOVA was performed. *, *p* < 0.05; **, *p* < 0.01. All values are shown as mean ± SEM. (DOC 609 kb)

Additional file 4: **Figure S2.** IFN-γ score is an efficient candidate for prognostic indicator of glioma. (A) The expression of PD-L1 and other IFN-γ-induced genes increases along with the malignancy degree of glioma based on the LGG/GBM TCGA datasets. OD, oligodendroglioma; OA, oligoastrocytoma; AST, astrocytoma; GBM, glioblastoma multiforme. (B) The expression of PD-L1 and other IFN-γ-induced genes was negatively correlated with the survival of glioma patients based on the LGG/GBM TCGA datasets. (DOC 1515 kb)

## Abbreviations

BMDM: Bone marrow-derived macrophages
CNS: Central nervous system
CT: Cellular tumor
CTpan: Pseudopalisading cells around necrosis
CTpnz: Perinecrotic zone
FACS: Fluorescence-activated cell sorting
GBM: Glioblastoma
IHC: Immunohistochemistry
IM: Invasive margin
IT: Intratumoral region
LGG: Lower grade glioma
N: Normal tissue
TCGA: The Cancer Genome Atlas
TR: Tumor rim
