# Supplementary material for: The IFN-γ/PD-L1 axis between T cells and tumor microenvironment: hints for glioma anti-PD-1/PD-L1 therapy
Source: J Neuroinflammation. 2018 Oct 17;15:290. doi: 10.1186/s12974-018-1330-2 (PMC6192101; doi:10.1186/s12974-018-1330-2)
Supplement: Supplementary file 1 — Table S2. Technical specifications of antibodies used in our study. (DOC 46 kb) [file 12974_2018_1330_MOESM1_ESM.doc]

Additional file 1: **Table S2.** Technical specifications of antibodies used in our study

| **Antigen** | **Conjugation** | **Clone** | **Source** | **Cat.No.** | **App** |
| --- | --- | --- | --- | --- | --- |
| AnnexinV | APC | - | BD | 550474 | FC |
| CD3e | FITC | 17A2 | eBioscience | 11-0032 | FC |
| CD4 | PB | GK1.5 | eBioscience | 48-0041 | FC |
| CD8 | FITC | 53-6.7 | eBioscience | 11-0081 | FC |
| CD8 | APC | 53-6.7 | eBioscience | 17-0081 | FC |
| IFN-γ | PE | XMG1.2 | eBioscience | 12-7311 | FC |
| CD45 | PE-Cy7 | 30-F11 | eBioscience | 25-0451 | FC |
| CD45 | PB | 30-F11 | eBioscience | 48-0451 | FC |
| CD45 | APC | 30-F11 | eBioscience | 17-0451 | FC |
| CD11b | eFluor 450 | M1/70 | eBioscience | 48-0112 | FC |
| CD11b | FITC | M1/70 | eBioscience | 11-0112 | FC |
| PD-1 | PE | J43 | eBioscience | 12-9985 | FC |
| PD-1 | PE-Cy7 | J43 | eBioscience | 25-9985 | FC |
| PD-L1 | PE | MIH5 | BD | 558091 | FC |
| PD-L1 | PE-Cy7 | MIH5 | eBioscience | 25-5982 | FC |
| CD4 | Biotin | GK1.5 | R&D | BAM554 | IF |
| CD8 | Biotin | 53-6.7 | eBioscience | 13-0081 | IF |
| Iba1 | - | - | Wako | 019-19741 | IF |
| PD-L1 | Biotin | MIH5 | eBioscience | 13-5982 | IF |
