# Supplementary material for: The IFN-γ/PD-L1 axis between T cells and tumor microenvironment: hints for glioma anti-PD-1/PD-L1 therapy
Source: J Neuroinflammation. 2018 Oct 17;15:290. doi: 10.1186/s12974-018-1330-2 (PMC6192101; doi:10.1186/s12974-018-1330-2)
Supplement: Supplementary file 2 — Table S1. Primer sequences for qPCR used in the study. (DOC 31 kb) [file 12974_2018_1330_MOESM2_ESM.doc]

**Additional file 2: Table S1. Primer sequences for qPCR used in the study**

| **Gene** | **Forward (5'to3')** | **Reverse (5'to3')** |
| --- | --- | --- |
| *Irf1* | ATCTCGGGCATCTTTCGCTT | TGCATCTCTAGCCAGGGTCT |
| *Ifng* | AGGAACTGGCAAAAGGATGGT | TCATTGAATGCTTGGCGCTG |
| *Gbp5* | AGGTCAACGGACCTCGTCTA | CTATGGCCTCTCGCTCACAG |
| *Cd274* | GCTCCAAAGGACTTGTACGTG | TGATCTGAAGGGCAGCATTTC |
| *Ccl2* | GGGATCATCTTGCTGGTGAA | AGGTCCCTGTCATGCTTCTG |
| *Actin* | CGTGAAAAGACCCAGATCA | CACAGCCTGGATGGCTACGT |
