# Supplementary material for: The IFN-γ/PD-L1 axis between T cells and tumor microenvironment: hints for glioma anti-PD-1/PD-L1 therapy
Source: J Neuroinflammation. 2018 Oct 17;15:290. doi: 10.1186/s12974-018-1330-2 (PMC6192101; doi:10.1186/s12974-018-1330-2)
Supplement: Supplementary file 3 — Figure S1. Distribution of PD-L1 in the murine glioma model. (A) Representative staining for PD-L1 (red) and DAPI (blue) in the tumor-bearing brain (day 20); different regions of the tumor-bearing brain were shown. Scale bar, 100 μm. (B) PD-L1 intensity of different regions was calculated. Data were collected from four to seven randomly selected areas for each corresponding region per mouse, n = 3. One-way ANOVA was performed. *, p < 0.05; **, p < 0.01. All values are shown as mean ± SEM. (DOC 609 kb) [file 12974_2018_1330_MOESM3_ESM.doc]

Additional file 3

**
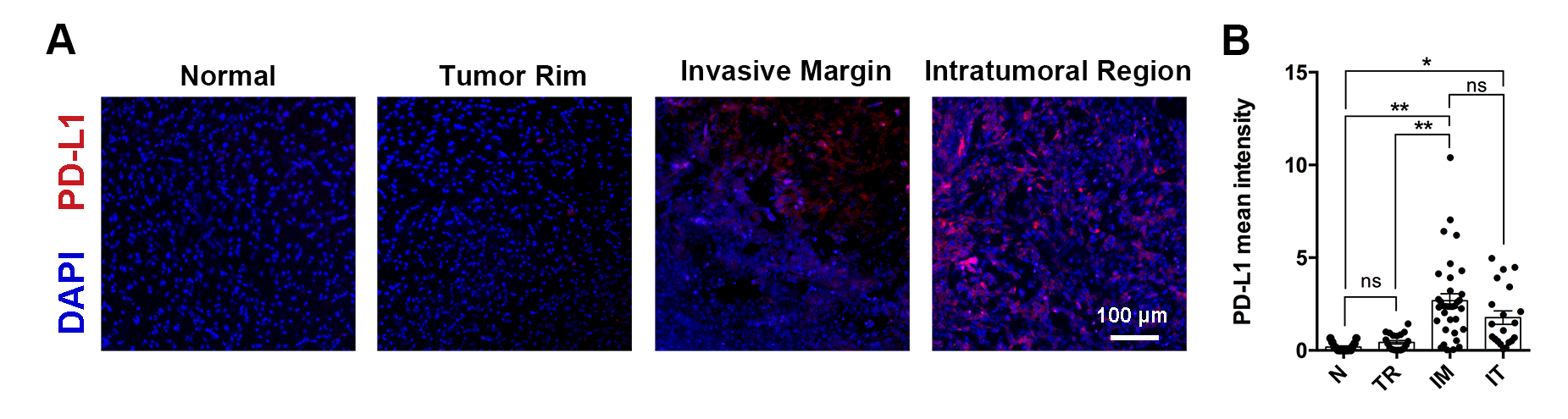
**

**Figure S1. Distribution of PD-L1 in murine glioma model (A)** Representative staining for PD-L1 (red) and DAPI (blue) in the tumor-bearing brain (Day 20), different regions of the tumor-bearing brain were shown. Scale bar, 100 μm. **(B)** PD-L1 intensity of different regions was calculated. Data were collected from 4-7 randomly selected areas for each corresponding region per mouse, n = 3. One-way ANOVA was performed. *, P < 0.05; **, P < 0.01. All values are shown as mean ± SEM.
