# Supplementary material for: The IFN-γ/PD-L1 axis between T cells and tumor microenvironment: hints for glioma anti-PD-1/PD-L1 therapy
Source: J Neuroinflammation. 2018 Oct 17;15:290. doi: 10.1186/s12974-018-1330-2 (PMC6192101; doi:10.1186/s12974-018-1330-2)
Supplement: Supplementary file 4 — Figure S2. IFN-γ score is an efficient candidate for prognostic indicator of glioma. (A) The expression of PD-L1 and other IFN-γ-induced genes increases along with the malignancy degree of glioma based on the LGG/GBM TCGA datasets. OD, oligodendroglioma; OA, oligoastrocytoma; AST, astrocytoma; GBM, glioblastoma multiforme. (B) The expression of PD-L1 and other IFN-γ-induced genes was negatively correlated with the survival of glioma patients based on the LGG/GBM TCGA datasets. (DOC 1515 kb) [file 12974_2018_1330_MOESM4_ESM.doc]

Additional file 4

**
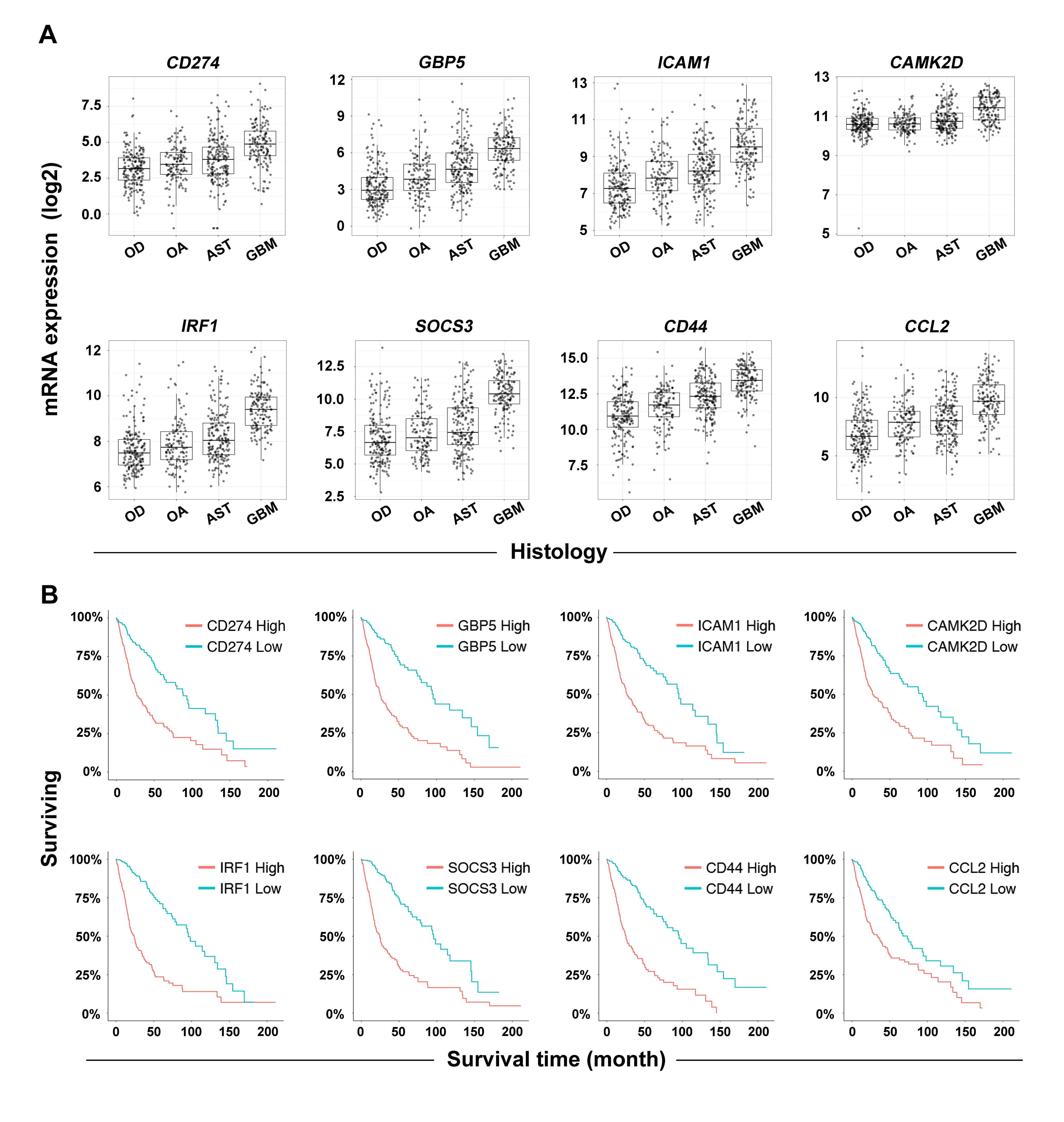
**

**Figure S2.** **IFN-γ score is an efficient candidate for prognostic indicator of glioma (A)** The expression of PD-L1 and other IFN-γ induced genes increases along with the malignancy degree of glioma based on the LGG/GBM TCGA datasets. OD, oligodendroglioma; OA, oligoastrocytoma; AST, astrocytoma; GBM, glioblastoma multiforme. **(B)** The expression of PD-L1 and other IFN-γ induced genes was negatively correlated with the survival of glioma patients based on the LGG/GBM TCGA datasets.
